# New Model for Gastroenteropancreatic Large-Cell Neuroendocrine Carcinoma: Establishment of Two Clinically Relevant Cell Lines

**DOI:** 10.1371/journal.pone.0088713

**Published:** 2014-02-14

**Authors:** Andreas Krieg, Sabrina Mersch, Inga Boeck, Levent Dizdar, Eberhard Weihe, Zena Hilal, Markus Krausch, Birte Möhlendick, Stefan A. Topp, Roland P. Piekorz, Wolfgang Huckenbeck, Nikolas H. Stoecklein, Martin Anlauf, Wolfram T. Knoefel

**Affiliations:** 1 Department of Surgery (A), Heinrich-Heine-University and University Hospital Duesseldorf, Duesseldorf, Germany; 2 Institute of Pathology, Heinrich-Heine-University and University Hospital Duesseldorf, Duesseldorf, Germany; 3 Institute of Anatomy and Cell Biology, Department of Molecular Neuroscience, Philipps University Marburg, Marburg, Germany; 4 Institute of Biochemistry and Molecular Biology II, Heinrich-Heine University Duesseldorf, Duesseldorf, Germany; 5 Institute of Forensic Medicine, Heinrich-Heine-University and University Hospital Duesseldorf, Duesseldorf, Germany; National Cancer Institute, United States of America

## Abstract

Recently, a novel WHO-classification has been introduced that divided gastroenteropancreatic neuroendocrine neoplasms (GEP-NEN) according to their proliferation index into G1- or G2-neuroendocrine tumors (NET) and poorly differentiated small-cell or large-cell G3-neuroendocrine carcinomas (NEC). Our knowledge on primary NECs of the GEP-system is limited due to the rarity of these tumors and chemotherapeutic concepts of highly aggressive NEC do not provide convincing results. The aim of this study was to establish a reliable cell line model for NEC that could be helpful in identifying novel druggable molecular targets. Cell lines were established from liver (NEC-DUE1) or lymph node metastases (NEC-DUE2) from large cell NECs of the gastroesophageal junction and the large intestine, respectively. Morphological characteristics and expression of neuroendocrine markers were extensively analyzed. Chromosomal aberrations were mapped by array comparative genomic hybridization and DNA profiling was analyzed by DNA fingerprinting. *In vitro* and *in vivo* tumorigenicity was evaluated and the sensitivity against chemotherapeutic agents assessed. Both cell lines exhibited typical morphological and molecular features of large cell NEC. *In vitro* and *in vivo* experiments demonstrated that both cell lines retained their malignant properties. Whereas NEC-DUE1 and -DUE2 were resistant to chemotherapeutic drugs such as cisplatin, etoposide and oxaliplatin, a high sensitivity to 5-fluorouracil was observed for the NEC-DUE1 cell line. Taken together, we established and characterized the first GEP large-cell NEC cell lines that might serve as a helpful tool not only to understand the biology of these tumors, but also to establish novel targeted therapies in a preclinical setup.

## Introduction

Gastroenteropancreatic neuroendocrine neoplasms (GEP-NEN) represent a rare, morphologically homogeneous, however biologically and clinically very heterogeneous group of tumors originating from the diffuse neuroendocrine cell system. According to the WHO classification they are characterized by the expression of general neuroendocrine vesicle marker proteins, i.e. chromogranin A (*CGA*) and synapthophysin (*SYN*) [1], [2], [3], [4], [5]. Depending on their anatomic site of origin, NENs can be classified into foregut, midgut and hindgut tumors [6]. The revised WHO-classification from 2010 integrated the recommendations of the European Neuroendocrine Tumor Society (ENETS) and categorizes NENs according to their proliferative activity into (well differentiated) neuroendocrine tumors (NET) grade 1 (G1: <2 mitoses/10 high power fields; Ki-67 index ≤2%), moderately differentiated grade 2 tumors (G2: 2–20 mitoses/10 high power fields; Ki-67 index 3–20%) and poorly differentiated and clinically highly aggressive grade 3 large cell or small cell type neuroendocrine carcinomas (NEC; G3: >20 mitoses/10 high power fields; Ki-67 index >20%) [7], [8].

Approximately 70% of patients with NENs present at the time of diagnosis with advanced, metastatic disease [9]. According to recently published guidelines resection with curative intend using standard oncological principles is the first line therapy for patients with limited disease [8], [10]. However, if extensive metastatic disease has occurred, interdisciplinary therapeutic approaches might be feasible including surgical debulking, interventional embolization techniques, radiofrequency ablation or chemotherapy [10]. Chemotherapeutic drugs such as streptozotocin, 5-fluorouracil (5-FU), cisplatin and etoposide or targeted agents inhibiting growth factor receptors, tyrosine kinases, mTOR signaling as well as somatostatin receptor antagonists provide effective treatments only in distinct subpopulations of NENs [11], [12].

However, for highly malignant GEP-NECs that are associated with a very poor prognosis, for a limited disease without distant metastasis the first line treatment consists of curative surgical resection using standard oncological criteria [13], [14]. Systemic chemotherapy with platinum-based drugs and etoposide is indicated for progressive and metastasized GEP-NECs and may be considered only in a subset of cases as adjuvant therapy [13], [14], [15]. However, response rates are low and, although alternative chemotherapeutic concepts with oxaliplatin and 5-fluorouracil or capecitabine have been reported, there is so far no established second line therapy [16], [17], [18], [19], [20].

Gaining insights in the biology of large cell NECs is crucial for the identification of potentially therapeutic molecular targets. For this purpose, cell lines derived from tumor tissue specimens provide helpful tools [21]. Although during the last decades a few gastrointestinal NEN cell lines have been established, a major problem is the heterogeneous pathological terminology when trying to classify these cell lines according to the revised WHO classification with respect to their original tumors [22], [23], [24], [25], [26], [27], [28], [29]. In addition, to our knowledge the establishment of defined large-cell GEP-NEC cell lines has not been reported yet [30]. The lack of a well characterized and reliable cell culture model for NECs led us to the establishment of two novel large-cell NEC cell lines originating from NECs of the gastroesophageal junction and the large intestine. Both cell lines, designated NEC-DUE1 and -DUE2, were characterized by electron microscopy and expression profiling with general and specific neuroendocrine marker proteins and were tested in cell culture and *in vivo* for tumorigenicity and metastatic properties.

## Materials and Methods

### Tissue Sample Processing, Cell Lines and Cell Culture

This study was approved by the ethics committee of the Medical Faculty, Heinrich Heine University Duesseldorf (study number: 3457), and patients gave written informed consent. Immediately after surgical resection, tumor tissue fragments measuring up to 0.5 cm in diameter were mechanically disassociated for up to 2 minutes in 1 ml RPMI using the Medimachine System (BD Biosciences, Heidelberg, Germany). The cell suspension was recovered from the disaggregator and cultured in 6 well plates in a final volume of 2 ml RPMI medium (Gibco, Karlsruhe, Germany) supplemented with 10% heat inactivated FCS (fetal calf serum), penicillin and streptomycin at 37°C in an atmosphere with 5% CO_2_.

In addition to the established cell lines, human colon cancer cell line HCT116 obtained from the American Type Culture Collection (ATCC, LGC Standards GmbH, Wesel, Germany) served in some experiments as control and was cultured in McCoy’s 5A medium supplemented with 10% FCS. NEC cell lines were permanently maintained in RPMI medium supplemented with 10% heat inactivated FCS, penicillin and streptomycin at 37°C in an atmosphere with 5% CO_2_.

### 3-Dimensional (3D)-cell Culture

3-Dimensional (3D) cell cultures were prepared within growth factor reduced laminin-rich extracellular matrix (lrECM 3D) as recently described [31]. Therefore, 120 µl matrigel (BioCoat Matrigel Basement Membrane, BD Biosciences) per 24 well was plated and incubated for 15 minutes at 37°C and 5% CO_2_. 1.8×10^4^ cells were resuspended in 250 µl culture medium, plated into the matrigel coated wells and incubated for additional 15 minutes at 37°C and 5% CO_2_. Subsequently, 225 µl culture medium supplemented with 10% matrigel was added to the wells. The cells were cultured for seven days under standard conditions. Medium containing 10% matrigel was changed every second day. Cell recovery was performed by adding dispase (BD Biosciences) to dissolve the matrigel matrix. The reaction was stopped by adding EDTA/PBS. Spheroids were obtained after accumulation at the bottom of the culture vessel, applied to microscope slides and air dried overnight. The experiment was performed in duplicates and was compared to 2-dimensional (2D) culture conditions.

### Immunofluorescence Staining of Fixed Spheres

Spheroids were fixed and washed in 1×PBS. Unspecific binding sites were blocked for 20 minutes with 5% milk/TBS-T. Primary antibody beta-Actin (Sigma-Aldrich, Hamburg, Germany) was diluted 1∶5000 in 5% milk/TBS-T and incubated overnight at 4°C. Subsequently, cells were washed in 1×PBS and incubated with 10 µg/ml secondary Alexa Fluor 488 goat anti mouse IgG antibody (Invitrogen/Life Technologies, Darmstadt, Germany) for 60 minutes in the dark. Counterstaining of nuclear DNA was performed with 0.01 µg/ml DAPI (4′,6-Diamidin-2-phenylindol; Sigma-Aldrich) in 1×PBS for four minutes at room temperature. After washing twice with 1×PBS, spheroids were mounted with Vectashield Mounting Medium. Imaging was done by using LSM510-Meta confocal laser scanning microscope (Zeiss, Jena, Germany) with a 40x/1.3 immersion objective.

### Immunocytochemistry and Immunohistochemistry

Cells were grown overnight on cover slips and fixed with methanol and acetone. Tissue sections (2 µm) were deparaffinised and rehydrated. Endogenous peroxidase activity was blocked with 0.3% hydrogen peroxide. Blocking non-specific protein-binding sites, normal mouse serum was applied. Neuroendocrine marker proteins were detected by specific antibodies as summarized in **Table S1**. Immunostaining was performed with anti-mouse and anti-rabbit IgG and Vectastatin ABC kit (Vector Lab, Burlingame, CA, USA) followed by chromogen detection as described previously [32].

### Electron Microscopy

Adherent cells were scrapped from the culture dishes. Subsequently, cells were centrifuged for 10 min and fixed with 4% paraformaledehyde and 0.3% glutaraldehyde. Fixed cells were washed, dehydrated in acetone and embedded in LRWhite resin (Sigma-Aldrich). Thin sections (50–70 nm) were collected on nickel grids and stained with uranyl acetate and lead citrate. Imaging was performed by using the EM 109 R electron microscope (Zeiss).

### RNA Isolation and RT-PCR Analysis

Total RNA from cell lines was isolated using the RNeasy Mini Kit (Qiagen, Hilden, Germany) according to the manufacturer’s protocol. Synthesis of cDNA was performed by reverse transcription with 0.025 µg oligo-d(T)-primer (Invitrogen/Life Technologies) and Transcriptor Reverse Trancriptase (Roche, Mannheim, Germany) in a final volume of 20 µl.

For amplification 50 ng cDNA was diluted in a final volume of 50 µl containing 25 µl Dream Taq Green PCR Master Mix (Thermo Scientific, Waltham, MA, USA) and 5 µl Primer mix (8 µM each). The PCR program was started with an initial denaturation step at 95°C for 2 minutes followed by 29 cycles including 30 seconds at 95°C, 30 seconds at 52°C and 12 seconds at 72°C. Final elongation was performed at 72°C for 2 minutes. Primer sequences for PCR are summarized in **Table S2**. The PCR products were separated in 2% agarose gels and detected using the Versa Doc system (Bio-RAD, Munich, Germany).

### DNA Preparation

Genomic DNA (gDNA) from the cell lines was prepared using the QIAamp DNA Blood Midi Kit (Qiagen) according to the manufacturer’s protocol. DNA from formalin-fixed, paraffin-embedded (FFPE) tissues was isolated utilizing the QIAamp DNA FFPE Tissue Kit (Qiagen). For this method, areas containing tumor tissue as well as normal tissue were separately macrodissected with a gauge needle from a microscope slide. DNA quality was checked on a 1.5% agarose gel and DNA concentration was determined using the Infinite® 200 PRO NanoQuant spectrometer (Tecan Group Ltd., Crailsheim, Germany).

### Short Tandem Repeat (STR) Analysis

For DNA fingerprinting analysis, multiplex PCR reactions were performed by amplifying 1 ng of genomic DNA using the genRESH MPX-2 and genRESH MPX-3 kits (Serac GmbH, Bad Homburg, Germany). Amplified products were analyzed on an ABI 3100 capillary sequencer and profiled by the genotyper V3.10 software (Applied Biosystems, Carlsbad, CA, USA).

### Comparative Genomic Hybridization with Oligonucleotide Microarrays (aCGH)

Array CGH analyses on oligonucleotide arrays were performed according to the manufacturer’s instructions (Agilent Oligonucleotide Array-Based CGH for Genomic DNA Analysis, Version 7.1; Agilent Technologies, Santa Clara, CA, USA). Briefly, 500 ng gDNA from each cell line as well as pooled normal Megapool Reference DNA (Kreatech, Amsterdam, Netherlands) were digested with AluI and RsaI. The digestion step was skipped for the FPPE samples, which provided optimal fragment sizes for successive labeling. Random-primed labeling (RP) was performed with the Genomic DNA Enzymatic Labeling Kit (Agilent Technologies) according to the manufacturer’s protocol (Version 7.1). The fluorescently labeled DNAs from the cell lines, as well as from the FFPE samples were hybridized to the 8×60 k platform. The oligonucleotide arrays were processed using the Microarray Scanner G2565CA by Agilent Technologies with 3 µm resolution and 16 bit color depth. The output image files were imported; normalized and fluorescent ratios for each probe were determined using Feature Extraction software (Agilent Technologies, Version 10.7.3.1, Protocol CGH_107_Sep09). Feature Extraction output files were imported into the Genomic Workbench 5.0.14 software. Array CGH data were examined using the aberration detection method 2 (ADM-2) algorithms with a threshold of 6.0. A custom aberration filter was defined for identifying copy number alterations. Changes were only considered as true positive events when characterized by a minimum log_2_ratio of ±0.25 and a minimum of three consecutive probes with the same polarity per region, reaching a resolution of ∼125 kb.

### Colony Formation Assay

In a 35 mm plate, 1.5 ml of a base layer was prepared by adding a sterile 2% agarose solution to 2×RPMI-media supplemented with 20% FCS in an 1∶1 ratio. During solidification at room temperature, harvested cells were adjusted to a concentration of 1×10^4^ cells per well in 2 ml of 1×RPMI supplemented with 10% FCS. Next, this cell suspension was added to a 1.5% top layer agarose solubilized in 2×RPMI supplemented with 20% FCS in an 1∶1 ratio. Subsequently, 1.5 ml of this mixture was plated on the base layer and incubated for 45 minutes at room temperature to allow the top layer to solidify before plates were incubated at 37°C with an atmosphere of 5% CO_2_. After 13 days of incubation, the number of colonies was microscopically determined in 10 visual fields/well at a 10×0.22 magnification.

### Invasion, Migration Assay and Tumor Xenograft Model

Invasion and migration chambers (BD Biosciences) were thawed to room temperature and rehydrated for two hours with culture medium containing 1% FCS at 37°C. The medium was removed and chamber inserts coated with matrigel were transferred to wells with culture medium containing 5% FCS. 1.5×10^4^ cells per insert were seeded in triplicates and incubated for 24 hours at 37°C and 5% CO_2_. The next day medium was removed and cells were fixed in 100% methanol for 10 minutes at room temperature. Subsequently cells were stained with crystal violet for 10 to 15 minutes. Staining solution was removed by washing the inserts with H_2_O. Non-invading and non-migratory cells on the inside of the filter membrane were eliminated by wiping the filter with a cotton swab. The membranes were placed on a microscope slide and covered with Entellan® (Merck, Darmstadt, Germany). Invasion or migration was quantified by counting cells in four visual fields of the membrane under an inverted light microscope (Leica DM IL; Leica, Wetzlar, Germany) with a 10×0.22 objective.

All animal experiments were performed in accordance with the regulations of German Law for the Protection of Animals and were evaluated and approved by the North-Rhine-Westfalian (NRW) Ministry for Environment and Nature Protection, Agriculture and Consumer Protection (Landesamt für Natur, Umwelt und Verbraucherschutz; LANUV NRW: 84-02.04.2011.A382). *In vivo* tumorigenicity was investigated by using a subcutaneous mouse xenograft model. NOD-scid IL2rgamma^null^ mice were bred in our animal facility under specific pathogen-free conditions. For each cell line 4 eight-week-old, female NOD-scid IL2rgamma^null^ mice were subcutaneously injected into the flank with 1×10^6^ of NEC-DUE1 or NEC-DUE2 cells resuspended in 100 µl of 1×PBS and mixed with 100 µl of matrigel. When tumors became palpable, mice were euthanized by CO_2_ inhalation. Subcutaneously growing tumors were excised and fixed in 10% formalin.

### Chemotherapeutic Drug Testing and Viability Assay

For chemotherapeutic treatment and viability assays, 2×10^4^ cells were seeded per well in 96 well plates. All chemotherapeutic agents were dissolved in DMSO. The next day cells were treated with cisplatin, etoposide, 5-FU or oxaliplatin (all Sigma-Aldrich) at a final concentration of 0.01, 0.03., 0.1, 0.3, 1, 3 or 10 µM. DMSO at equimolar concentrations to the chemotherapeutic agents served as negative control. Twenty-four hours after incubation, cell viability was measured using the CellTiter 96® AQueous Non-Radioactive Cell Proliferation Assay (Promega, Madison, WI, USA). Therefore, 20 µl of 3-(4,5-dimethylthiazol-2-yl)-5-(3-carboxymethoxyphenyl)-2-(4-sulfophenyl)-2H-tetrazolium (MTS) and phenazine methosulfate solution were added to the culture medium and cells were incubated for two hours at 37°C and 5% CO_2_. The absorbance of the cell culture medium that is directly proportional to the number of viable cells was measured at 490 nm. All experiments were performed in triplicates.

## Results

### Origin of Cell Lines

NEC-DUE1 originated from one of two atypically resected liver metastasis (Ki-67 index: 80%) of a 71-year old, male Caucasian with a history of neoadjuvant chemotherapy with cisplatin/etoposide and extended gastrectomy for a pT3 N1 L1 V1 G3 large-cell NEC at the gastroesophageal junction. Because of tumor progression during the follow up with progressive liver metastasis, bone metastasis and a cutaneous metastasis, adjuvant chemotherapy was changed from cisplatin/etoposide to the FOLFOX4 (Folinic Acid-Fluorouracil-Oxaliplatin) scheme. Under this chemotherapeutic concept, the patient showed a stable disease and was still alive thirty-eight months after diagnosis.

NEC-DUE2 was isolated from a lymph node metastasis of a pT4a N2b M1a (LYM) L1 V1 Pn1 G3 (Ki-67 index: 80%) large-cell NEC located at the right colic flexure of a 71-year old, male Caucasian without a history of neoadjuvant chemotherapy. Surgical therapy included subtotal colectomy with ileo-sigmoideostomy, hemi-gastrectomy with gastro-jejunostomy, total pancreatectomy, splenectomy and systematic lymphadenectomy. Because of tumor progression, the patient died three months after an uneventful postoperative course.

**Figure 1:**
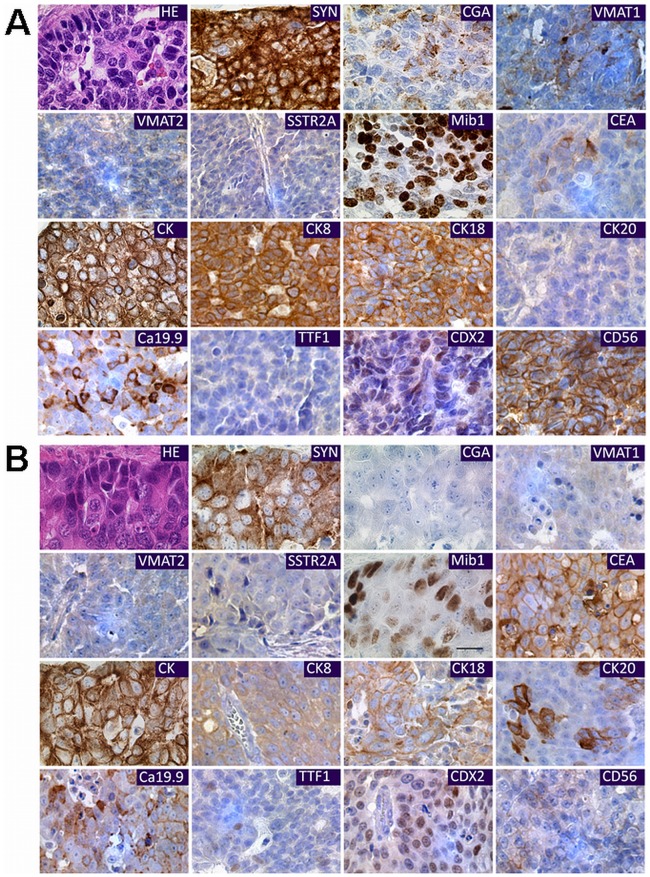
Morphological and immunohistochemical characterization of primary NECs. Primary tumors from which the cell lines NEC-DUE1 (**A**) and NEC-DUE2 (**B**) derived stained with hematoxylin-eosin (HE) showing morphologically large cell neuroendocrine cytology, i.e. large-sized cells with large atypical nuclei revealing a “salt and pepper” chromatin. Synaptophysin (SYN), chromogranin A (CGA), vesicular monoamine transporters (VMAT1, VMAT2), somatostatin receptor (SSTR2A), thyroid transcription factor 1 (TTF1), caudal type homeobox 2 (CDX2), cluster of differentiation 56 (CD56) and cytokeratins (CK) as well as epithelial markers (CEA, Ca19.9) were immunohistochemically evaluated as indicated and proliferation index is demonstrated by staining with MIB-1 antibody.

Primary tumors of both patients showed a large cell neuroendocrine cytology and stained immunohistochemically positive for distinct epithelial and neuroendocrine markers. In addition, the NEC located at the gastroesophageal junction displayed a positive staining for chromogranin A **(** **Figure 1** **)**.

### Morphological Characteristics and Neuroendocrine Marker Profile

**Figure 2:**
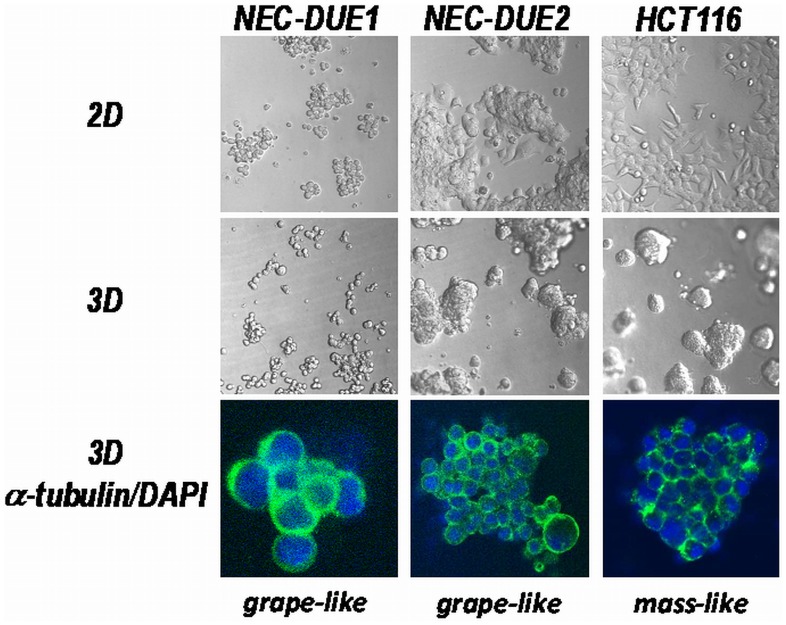
2D and 3D growth pattern of large-cell NEC cell lines. NEC-DUE1 and –DUE2 were cultivated in 2D (upper panel) or 3D culture systems (middle panel). The colon cancer cell line HCT116 served as control. Spheroids were grown in lrECM 3D microenvironments for seven days. Confocal laser scanning fluorescence microscopy images of isolated 3D spheroids (lower panel) stained with beta-actin (green) and DAPI (blue) revealed a grape-like growth pattern for both NEC cell lines.

Cell lines were growing as monolayer on conventional tissue culture plastic (2D) by forming colonies of round (NEC-DUE1) or polygonal (NEC-DUE2) cells **(** **Figure 2** **, upper panel)**. When cell lines were maintained in lrECM 3D on-top cultures **(** **Figure 2** **, middle panel)** and classified according to the 4 categories proposed by Kenny and colleagues [33], NEC-DUE1 cells displayed a specific morphology of colonies with poor cell-cell contacts and were therefore classified as grape-like spheroids. In contrast, NEC-DUE2 cells appeared by light microscopy to grow as cells of the mass-like class, however, visualization of the cytoskeleton by immunocytochemical staining of beta-actin **(** **Figure 2** **, lower panel)** clearly displayed the lack of stable cell-cell contacts. Thus, NEC-DUE2 cells were re-classified as cells from the grape-like morphology.

**Figure 3:**
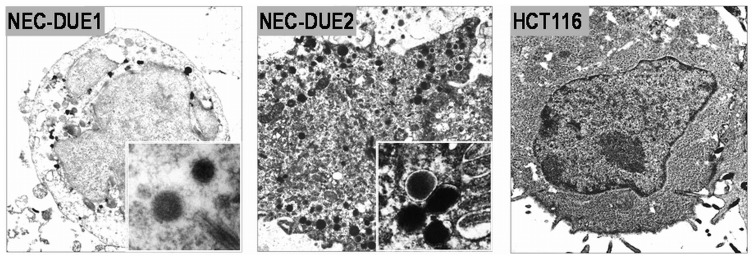
Electron microscopy of large-cell NEC cell lines. Electron microscopy revealed electron-dense large dense core neurosecretory granules in both NEC cell lines (inset demonstrates magnified electron dense granules). HCT116 served as negative control cell line.

One typical morphological hallmark of neuroendocrine cells is the presence of electron dense neurosecretory granules that store peptides and hormones [34]. Thus, we performed electron microscopy of both cell lines, NEC-DUE1 and -DUE2 and included epithelial colon cancer cell line HCT116 as negative control. In contrast to HCT116 cells as we expected, NEC-DUE1 and NEC-DUE2 cells exhibited the electron-dense cytoplasmic large dense core neurosecretory granula which are typical of neuroendocrine cells **(** **Figure 3** **)**.

**Figure 4:**
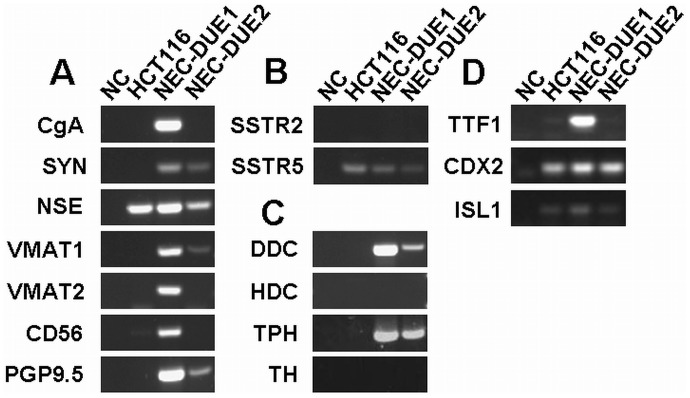
NEC cell lines express general and specific neuroendocrine markers, somatostatin receptors and transcription factors. RNA from cultured NEC cell lines was isolated and RT-PCR analyses performed for (**A**) general neuroendocrine markers, (**B**) somatostatin receptors, (**C**) specific neuroendocrine markers and (**D**) transcription factors as indicated. The colon cancer cell line HCT116 served as control cell line.

To further investigate the expression of neuroendocrine markers [35], we first performed RT-PCR analyses **(** **Figure 4** **)**. In contrast to HCT116 that served again as control, transcripts for neuroendocrine markers such as chromogranin A (*CGA)*, neuron specific enolase (*NSE)*, synaptophysin (*SYN)*, vesicular monoamine transporter 1 or 2 (*VMAT1*; *VMAT2)*, cluster of differentiation 56 (*CD56)* and gene product 9.5 (*PGP9.5)* were expressed in NEC-DUE1 **(** **Figure 4** **A)**. NEC-DUE2 expressed *SYN*, *NSE*, *VMAT1* and *PGP9.5* transcripts, although to a lower extent as NEC-DUE1. In contrast to somatostatin receptor 5 (SSTR5), SSTR2 mRNA was undetectable in the investigated cell lines **(** **Figure 4** **B)**. The specific neuroendocrine markers dopamine decarboxylase (*DDC*) and tryptophan hydroxylase 1 (*TPH1*) were only expressed in the NEC cell lines but not in HCT116 **(** **Figure 4** **C)**. Whereas the transcription factors thyroid transcription tactor (*TTF1*), caudal type homeobox 2 (*CDX2*) and islet-1 (*ISL1*) were detectable on mRNA levels in NEC-DUE1 cells, NEC-DUE2 expressed only CDX2 transcripts **(** **Figure 4** **D)**. Immunocytochemical expression profiles of both NEC-DUE1 and -DUE2 cells were comparable to the originate tumor and are summarized in **Table 1** **and Figure S1 A and B**.

**Table 1:** Immunohistochemical expression analyses of the cell lines NEC-DUE1 and NEC-DUE2.

	NEC-DUE 1		NEC-DUE2	
Antigen	*staining intensity*	*No. of cells (%)*	*staining intensity*	*No. of cells (%)*
***General neuroendocrine markers***				
Synaptophysin	+++	80	+++	80
Chromogranin A	+	30–40	–	0
VMAT 1	+	60	++	70
VMAT 2	–	0	–	0
CD56/NCAM	+++	100	++	80
***Somatostatin receptors***				
SSTR 2A	–	0	–	0
SSTR 5	–	0	–	0
***Proliferation***				
Ki-67	+++	70	+++	30–40
***Cytokeratins and epithelial markers***				
Pan-CK	+++	100	++	90
CK 8	+++	100	+++	80
CK 18	+++	100	+++	100
CK 20	–	0	–	0
CEA	+	10	+++	100
Ca 19.9	+++	20–30	++	80
***Transcription factors***				
TTF1	–	0	–	0
CDX2	–	0	++	70

NSE neuron-specific enolase, VMAT vesicular monoamine transporter, CD56 cluster of differentiation 56, NCAM Neural Cell Adhesion Molecule, SSTR somatostatin receptor, CK cytokeratin, CEA carcinoembryonic antigen, Ca 19.9 carbohydrate antigen 19-9, TTF1 thyroid transcription factor 1, CDX2 caudal type homeobox 2.

**Figure 5:**
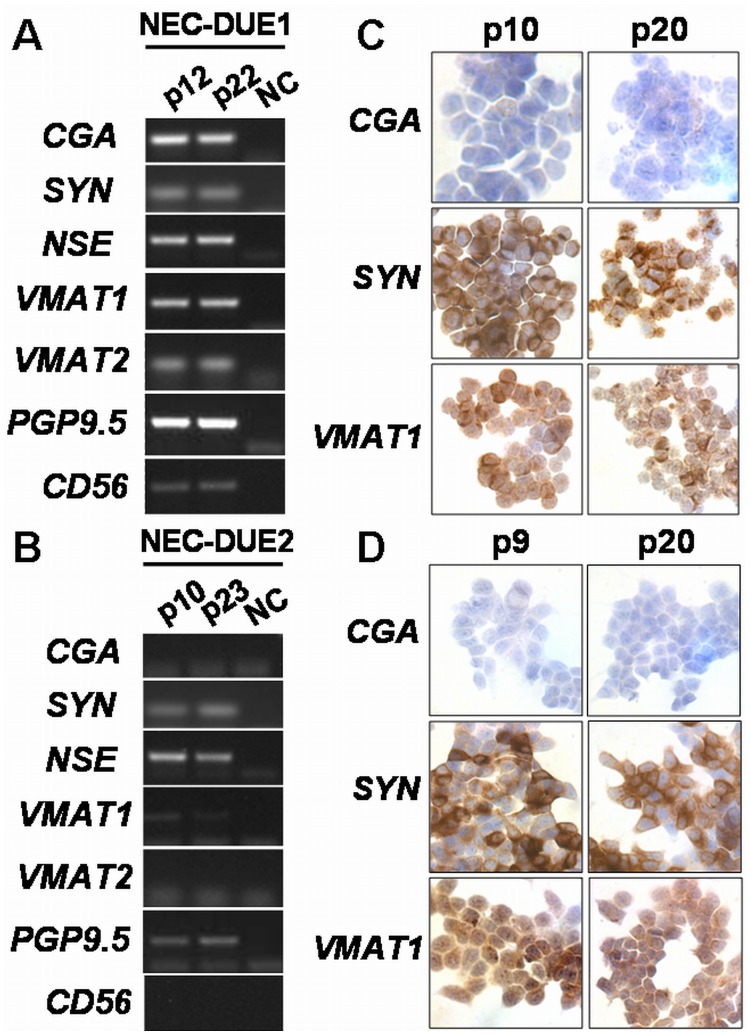
NEC-DUE1 and NEC-DUE2 retain the expression of neuroendocrine marker with increasing numbers of passage. RNA from cultured NEC cell lines NEC-DUE1 (**A**) and NEC-DUE2 (**B**) at indicated culture passages was isolated and RT-PCR analyses performed for neuroendocrine markers. Immunocytochemical staining of selected neuroendocrine markers was performed in NEC-DUE1 (**C**) and NEC-DUE2 (**D**) cells grown on cover slips at different numbers of culture passage. NC = negative control, p = number of passage.

Currently, both cell lines proliferate continuously over more than 25 passages since 2011. In addition, recovery of cryopreserved cells was uneventful. Importantly, during this period of time we did not observe any changes in morphology or growth pattern. However, to further provide evidence that these cells are viable neuroendocrine cell lines retaining their neuroendocrine expression profile, we analyzed changes in neuroendocrine marker profiles by RT-PCR **(** **Figure 5** **A and B)** and immunocytochemistry (**Figure 5** **C and D)** at different passages. Accordingly, a change in neuroendocrine expression with passage became not evident in both NEC-DUE1 and NEC-DUE2 cell lines.

### Cytogenetic Characterization

**Figure 6:**
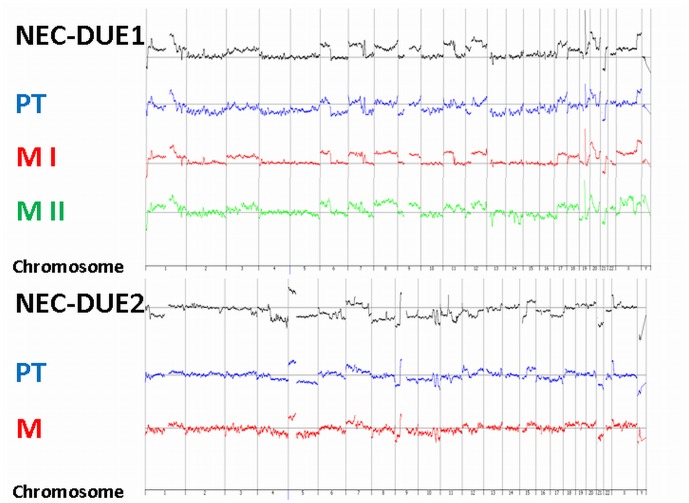
Cytogenetic changes in large-cell NEC cell lines. DNA was isolated from the cell lines, primary tumors (PT) and hepatic or lymphatic metastases (M) and genetic aberrations were analyzed by aCGH analysis. Amplitudes over the midline reflect chromosomal gains, amplitudes under the midline losses. M I and M II represent the atypically resected liver metastases of the gastroesophageal NEC.

First, we confirmed the identity of the established cell lines by STR-analysis comparing specific regions on the DNA from both the cell line and the patient’s tumor **(Table S3)**. We then performed aCGH analyses to characterize cytogenetic changes in primary tumors, metastases and cell lines. Accordingly, DNA samples from NEC-DUE1 and -DUE2 exhibited highly similar genetic alterations when compared with the primary tumor and metastases, respectively. Thus, aCGH confirmed the origin of each cell line by clearly showing cytogenetic matches with the original tumor **(** **Figure 6** **)**. Whereas NEC-DUE1 was characterized by a higher number of chromosomal gains than losses, we observed more losses than gains of chromosomal material in NEC-DUE2. The most common genetic gains in both NEC cell lines were localized on chromosomes 1q, 2p, 7p, 11p, 11q, 12p, 15q, 20q, and Xp, whereas the most frequent deletions were observed on chromosome 21q.

### Tumorigenicity *in vitro* and *in vivo*

**Figure 7:**
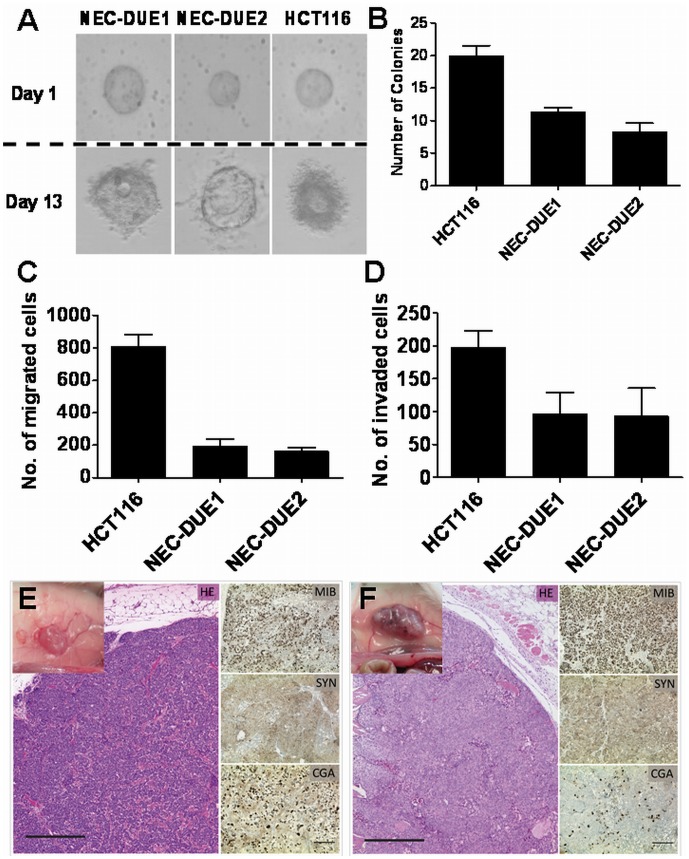
In vitro and in-DUE1 and -DUE2 cell lines. *In vitro* tumorigenicity was investigated by evaluating anchorage-independent growth in a colony formation assay. After 13 days visible colonies (**A**) were quantified (**B**). Migration (**C**) and invasion (**D**) were evaluated in a Boyden chamber assay. HCT116 served in all experiments as positive control cell line. Data represent means ± SD of three independent experiments. *In vivo* tumorigenicity was tested in a mouse model. Four weeks after injection of NEC-DUE1 (**E**) or NEC-DUE2 (**F**) tumor cells into the flank of immunocompromised mice tumor nodules were palpable. Mice were sacrified and tumor sections were stained with hematoxylin-eosin (HE) and analyzed for the expression of chromogranin A (CGA), synaptophysin (SYN) and Ki-67. Long scale bar = 500 µm; short scale bar = 200 µm.

*In vitro* tumorigenicity was first tested by evaluating anchorage-independent growth which is typical observed in malignant cells. Therefore, we performed colony formation assays by cultivating cells in soft agar culture systems. The colon cancer cell line HCT116 known to have a malignant phenotype both *in vitro* and *in vivo* served as control. Although HCT116 cells formed nearly 2-fold more colonies 13 days after cells were plated, both NEC-DUE1 and -DUE2 were able to grow under this semisolid culture condition that is typically observed for malignant cells **(** **Figure 7** **A and B)**. To further support these observations, we explored the migratory and invasive capacity of NEC cell lines. To analyze these malignant properties, we investigated cell migration in a Boyden chamber as well as cellular invasion capacity by employing Matrigel coated chambers. Taken together, these experiments revealed that both NEC-DUE1 and -DUE2 cells display a clear migratory and particularly invasive capacity *in vitro* **(** **Figure 7** **C and D)**.

In addition, *in vivo* tumorigenicity was evaluated in a xenograft model by injecting 1×10^6^ cells subcutaneously into the flank of four immunocompromised mice per cell line. Whereas one mouse of the NEC-DUE2 group passed away during the period of observation without any visible tumor burden, tumor nodules were palpable in all remaining mice after 20 days. At day 24, mice were sacrificed and tumors assessed for further morphological and immunohistochemical characteristics. As shown in **Figure 7** **E and F** both NEC-DUE1 and -DUE2 formed tumors that exhibited macroscopically as well as microscopically a dense neovascularization with a high Ki-67 proliferation index and positive staining for neuroendocrine markers that was consistent with the marker profile observed in the primary tumors.

### Distinct Sensitivity to Chemotherapeutic Drugs

**Figure 8:**
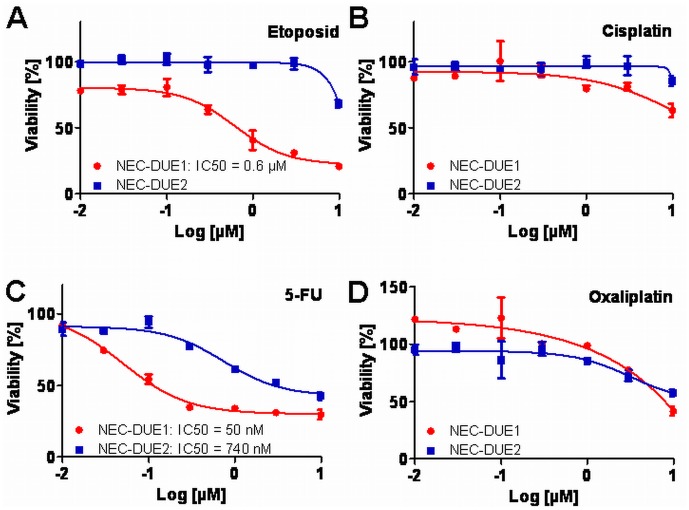
Sensitivity of NEC cell lines to conventional chemotherapeutics. NEC-DUE1 and –DUE2 were treated for 24 hours with etoposide (**A**), cisplatin (**B**), 5-FU (**C**) or oxaliplatin (**D**). Cell viability was measured using the MTS assay as described in materials and methods. Values represent the mean absorbance at 490 nm ± SD of triplicates.

Currently, first line chemotherapeutic concepts for advanced G3 GEP-NECs exhibiting distant metastases favor a combinatorial therapy including cisplatin and etoposide [13], [14], [15]. Thus, we incubated both NEC-DUE1 and -DUE2 with increasing concentrations of these chemotherapeutic agents. Whereas only NEC-DUE1 showed only a moderate drug response when treated with etoposide with a relatively high IC50 value **(** **Figure 8** **A)**, drug response curves of both cell lines incubated with cisplatin did not exhibit the typical S-shaped curve observed for drug sensitive cells **(** **Figure 8** **B)**. Since the patient from whom we isolated and established NEC-DUE1 was in the condition of a stable disease under the chemotherapy according to the FOLFOX4 concept, we additionally incubated both NEC cell lines with 5-FU and oxaliplatin. Interestingly, viability of NEC-DUE1 and NEC-DUE2 cells was characterized by a dose dependent decrease when treated with increasing concentrations of 5-FU **(** **Figure 8** **C)**. However, NEC-DUE1 was more sensitive to 5-FU with a low IC50 of 50 nM when compared to NEC-DUE2. In contrast, we observed a high resistance against oxaliplatin for both NEC-DUE1 and NEC-DUE2 **(** **Figure 8** **D)**.

## Discussion

Although the incidence of GEP-NEN increased during the last decades, based on the latest WHO classification they are still considered to be a rare tumor entity with an estimated incidence of 2.51/100 000 [9].

As reported by Modlin and colleagues, the major site of GEP-NENs is the intestine, followed by rectum and stomach [36]. Importantly, 15% of the gastric NENs with malignant biological behavior and approximately 29% of the colonic NENs are poorly differentiated NECs [9]. In contrast to the well differentiated NETs, the subgroup of poorly differentiated NECs, which was established in the revised WHO classification 2010, is characterized by a higher frequency of distant metastases at initial diagnosis and is associated with an extremely poor survival. Thus, G3 NECs represent with 6.7% of all GEP-NENs a rare, but highly aggressive tumor entity that is associated with an extremely poor prognosis [9].

To date, the only curative therapeutic approach can be achieved by radical oncological surgery [10]. Chemotherapeutic concepts for the treatment of metastasized highly aggressive NECs still recommend combinatorial chemotherapy with cisplatin and etoposide, but demonstrate only frustrating results [12], [13], [14], [15], [16], [17], [18]. Therefore, for NECs it is highly important to develop new therapeutic strategies that improve the patients outcome. In this context, the existence of appropriate and reliable *in vitro* and *in vivo* experimental models is mandatory. One of the first steps in testing the efficiency of novel drugs is to evaluate the influence of these substances on cell viability, proliferation, apoptosis, colony formation, as well as migration and invasion in cell lines that have been established from primary or metastatic tumors. To date, only a very small number of GEP-NEN cell lines have been established, some of which are characterized insufficiently [22], [23], [24], [25], [26], [27], [29]. Most of them originate from ileal or pancreatic NENs, none of them from the stomach [30]. In addition, none of these published cell lines has been classified according to the proliferation-based grading of the latest WHO classification. Moreover, so far only three cell lines have been established from colorectal tumors with neuroendocrine features, but all of them exhibiting an uncertain differentiation [24], [26], [27].

To our knowledge, herein we report the establishment and accurate characterization of the first large cell GEP-NEC cell lines. Both cell lines, named NEC-DUE1 and NEC-DUE2, exhibited a typical neuroendocrine cytology and profile of markers that are commonly used in the characterization and diagnosis of neuroendocrine tumors. Whereas VMAT1 which coordinates the ATP-depending transport of monoamines between the cytoplasm and secretory vesicles, was detectable on mRNA levels in NEC-DUE1 and -DUE2 cells, only the gastric NEC-cell line expressed VMAT2, known to be characteristic for gastric enterochromaffine-like (ECL) cells of the stomach [37], [38], [39], [40], [41]. Although we detected VMAT2 only by the more sensitive RT-PCR method, this detail was clearly consistent with the tissue origin of our cell lines. In addition, ultrastructurally, both cell lines exhibited typical neurosecretory granules in which monoamines and/or peptide hormones are stored and furthermore expressed the homeobox transcription factor CDX2 that is routinely used as a marker for gastrointestinal differentiation [34], [42].

Molecular profiling of GEP-NET by using conventional CGH analyses, revealed an average of 2.9 genomic aberrations in well differentiated gastrointestinal NETs [43]. In our study both NEC-DUE1 and NEC-DUE2 exhibited a higher number of genetic changes which may reflect the more aggressive biological behavior of G3 NECs. We did not observe the reported partial or complete loss of chromosome 18 [44]. However, gains on chromosome 7p and 20q that we identified in both of our NEC cell lines have been frequently observed in studies investigating chromosomal aberrations in NENs [43], [45], [46], [47]. In line with previous studies, we detected chromosomal gains in NEC-DUE1 and -DUE2 more frequently than chromosomal losses [45], [46]. Interestingly, genomic alterations have been observed more frequently in advanced and metastasized well-differentiated gastrointestinal NETs [43].

Interestingly, in contrast to HCT116 which was established from a primary colon cancer, both NEC-DUE1 and NEC-DUE2 which were derived from metastases formed the grape-like growth pattern with loose cell-cell contacts when cultivated under lrECM 3D conditions. Accordingly, this growth pattern seems to be typically observed in cell lines which have been established from metastases rather than from the primary site of tumor [31], [33]. Thus, both cell lines do not only demonstrate the typical morphological and immunohistochemical markers of neuroendocrine tumors but also retain the morphological characteristics of cells from metastases when cultured in an appropriate 3D microenvironment.

In addition to the malignant properties, which both NEC-cell lines retained *in vitro* as well as *in vivo*, they displayed the typical aggressive and chemoresistant phenotype that has been observed for GEP-NEC. In contrast to the colonic NEC-cell line which was highly resistant to commonly used chemotherapeutics, NEC-DUE1 demonstrated only a significant reduction in cell viability when treated with low doses of 5-FU. More importantly, this *ex vivo* sensitivity correlated with the stable clinical course of the patient from whom we established NEC-DUE1 under 5-FU containing chemotherapy according to the FOLFOX4 scheme. These data once more support the reliability of these novel NEC cell lines as a helpful tool in understanding the biological behavior of GEP-NEC and as a useful *ex vivo* model for further molecular phenotyping and drug screening experiments.

During the last decades only a few neuroendocrine cell lines have been established from GEP-NEN, but some of them seem to be not very well characterized with respect to the novel WHO classification. Mostly, these cell lines have been described as carcinoid, a term that was typically used for highly differentiated neuroendocrine tumors and carcinomas in the past until the new WHO classification in 2010 restricted this term for G1 NETs. In addition, some varying opinions on the authenticity of distinct cell lines have been reported in the literature [29], [48] and none of these established cell lines exhibited the typical features of large-cell GEP-NECs or have been derived from a tumor of the gastroesophageal junction [30].

In summary, to our knowledge we report the establishment and precise characterization of the first GEP-NEC cell lines that have been isolated from large-cell NECs. Importantly, our data supported the neuroendocrine and metastatic background of these novel cell lines and demonstrate that these cell lines might serve as a reliable model system for researchers to investigate neuroendocrine tumor biology and to identify novel molecular targets in the treatment against highly aggressive neuroendocrine carcinomas.

## Supporting Information

Figure S1 **Immunocytochemical expression profile of NEC cell lines.** NEC-DUE1 **(A)** and NEC-DUE2 **(B)** cells were grown on cover slips and stained with antibodies against general neuroendocrine markers, somatostatin receptors, proliferation marker, cytokeratines and epithelial markers as well as transcription factors. Abbreviations are explained in Table 1.
(TIF)

Table S1 **Antibodies used for immunocytochemistry and immunohistochemistry.**
(DOC)

Table S2 **Primers used for RT-PCR.**
(DOC)

Table S3 **STR-analysis of established cell lines and corresponding primary tumors.**
(DOC)

## References

[1] Rindi G, Buffa R, Sessa F, Tortora O, Solcia E (1986) Chromogranin A, B and C immunoreactivities of mammalian endocrine cells. Distribution, distinction from costored hormones/prohormones and relationship with the argyrophil component of secretory granules. Histochemistry 85: 19–28. 3525472 10.1007/BF00508649

[2] Yao JC, Hassan M, Phan A, Dagohoy C, Leary C, et al (2008) One hundred years after “carcinoid”: epidemiology of and prognostic factors for neuroendocrine tumors in 35,825 cases in the United States. J Clin Oncol 26: 3063–3072. 18565894 10.1200/JCO.2007.15.4377

[3] Wiedenmann B, John M, Ahnert-Hilger G, Riecken EO (1998) Molecular and cell biological aspects of neuroendocrine tumors of the gastroenteropancreatic system. J Mol Med (Berl) 76: 637–647. 9725766 10.1007/s001090050261

[4] Bishop AE, Polak JM, Facer P, Ferri GL, Marangos PJ, et al (1982) Neuron specific enolase: a common marker for the endocrine cells and innervation of the gut and pancreas. Gastroenterology 83: 902–915. 7106520

[5] Buffa R, Rindi G, Sessa F, Gini A, Capella C, et al (1987) Synaptophysin immunoreactivity and small clear vesicles in neuroendocrine cells and related tumours. Mol Cell Probes 1: 367–381. 3134611 10.1016/0890-8508(87)90018-1

[6] Williams ED, Sandler M (1963) The classification of carcinoid tum ours. Lancet 1: 238–239. 14000847 10.1016/s0140-6736(63)90951-6

[7] Bosman FT, Caneiro F (2010) WHO classification of tumours of the digestive system: Lyon: IARC Press.

[8] Kloppel G, Couvelard A, Perren A, Komminoth P, McNicol AM, et al (2009) ENETS Consensus Guidelines for the Standards of Care in Neuroendocrine Tumors: towards a standardized approach to the diagnosis of gastroenteropancreatic neuroendocrine tumors and their prognostic stratification. Neuroendocrinology 90: 162–166. 19060454 10.1159/000182196

[9] Niederle MB, Hackl M, Kaserer K, Niederle B (2010) Gastroenteropancreatic neuroendocrine tumours: the current incidence and staging based on the WHO and European Neuroendocrine Tumour Society classification: an analysis based on prospectively collected parameters. Endocr Relat Cancer 17: 909–918. 20702725 10.1677/ERC-10-0152

[10] Schott M, Kloppel G, Raffel A, Saleh A, Knoefel WT, et al (2011) Neuroendocrine neoplasms of the gastrointestinal tract. Dtsch Arztebl Int 108: 305–312. 21629514 10.3238/arztebl.2011.0305 PMC3103981

[11] Turner NC, Strauss SJ, Sarker D, Gillmore R, Kirkwood A, et al (2010) Chemotherapy with 5-fluorouracil, cisplatin and streptozocin for neuroendocrine tumours. Br J Cancer 102: 1106–1112. 20234360 10.1038/sj.bjc.6605618 PMC2853102

[12] Oberg K (2012) Neuroendocrine tumors of the digestive tract: impact of new classifications and new agents on therapeutic approaches. Curr Opin Oncol 24: 433–440. 22510940 10.1097/CCO.0b013e328353d7ba

[13] Ahlman H, Nilsson O, McNicol AM, Ruszniewski P, Niederle B, et al (2008) Poorly-differentiated endocrine carcinomas of midgut and hindgut origin. Neuroendocrinology 87: 40–46. 17940332 10.1159/000109976

[14] Nilsson O, Van Cutsem E, Delle Fave G, Yao JC, Pavel ME, et al (2006) Poorly differentiated carcinomas of the foregut (gastric, duodenal and pancreatic). Neuroendocrinology 84: 212–215. 17312381 10.1159/000098013

[15] Pavel M, Baudin E, Couvelard A, Krenning E, Oberg K, et al (2012) ENETS Consensus Guidelines for the management of patients with liver and other distant metastases from neuroendocrine neoplasms of foregut, midgut, hindgut, and unknown primary. Neuroendocrinology 95: 157–176. 22262022 10.1159/000335597

[16] Mitry E, Baudin E, Ducreux M, Sabourin JC, Rufie P, et al (1999) Treatment of poorly differentiated neuroendocrine tumours with etoposide and cisplatin. Br J Cancer 81: 1351–1355. 10604732 10.1038/sj.bjc.6690325 PMC2362979

[17] Moertel CG, Kvols LK, O’Connell MJ, Rubin J (1991) Treatment of neuroendocrine carcinomas with combined etoposide and cisplatin. Evidence of major therapeutic activity in the anaplastic variants of these neoplasms. Cancer 68: 227–232. 1712661 10.1002/1097-0142(19910715)68:2<227::aid-cncr2820680202>3.0.co;2-i

[18] Fazio N, Spada F, Giovannini M (2013) Chemotherapy in gastroenteropancreatic (GEP) neuroendocrine carcinomas (NEC): a critical view. Cancer Treat Rev 39: 270–274. 22819619 10.1016/j.ctrv.2012.06.009

[19] Bajetta E, Catena L, Procopio G, De Dosso S, Bichisao E, et al (2007) Are capecitabine and oxaliplatin (XELOX) suitable treatments for progressing low-grade and high-grade neuroendocrine tumours? Cancer Chemother Pharmacol 59: 637–642. 16937105 10.1007/s00280-006-0306-6

[20] Pape U, Tiling N, Bartel C, Plöckinger U, Wiedenmann B (2006) Oxaliplatin plus 5-fluorouracil/folinic acid as palliative treatment for progressive malignant gastrointestinal neuroendocrine carcinomas. J Clin Oncol 24: 14074.

[21] Rockwell S (1980) In vivo-in vitro tumour cell lines: characteristics and limitations as models for human cancer. Br J Cancer Suppl 4: 118–122. PMC2149198 6932914

[22] Pfragner R, Wirnsberger G, Niederle B, Behmel A, Rinner I, et al (1996) Establishment of a continuous cell line from a human carcinoid of the small intestine (KRJ-I). Int J Oncol 8: 513–520. 21544390 10.3892/ijo.8.3.513

[23] Lundqvist M, Mark J, Funa K, Heldin NE, Morstyn G, et al (1991) Characterisation of a cell line (LCC-18) from a cultured human neuroendocrine-differentiated colonic carcinoma. Eur J Cancer 27: 1663–1668. 1782079 10.1016/0277-5379(91)90441-f

[24] Quinn LA, Moore GE, Morgan RT, Woods LK (1979) Cell lines from human colon carcinoma with unusual cell products, double minutes, and homogeneously staining regions. Cancer Res 39: 4914–4924. 498117

[25] Pfragner R, Behmel A, Hoger H, Beham A, Ingolic E, et al (2009) Establishment and characterization of three novel cell lines - P-STS, L-STS, H-STS - derived from a human metastatic midgut carcinoid. Anticancer Res 29: 1951–1961. 19528452

[26] Stilling GA, Zhang H, Ruebel KH, Leontovich AA, Jin L, et al (2007) Characterization of the functional and growth properties of cell lines established from ileal and rectal carcinoid tumors. Endocr Pathol 18: 223–232. 18247165 10.1007/s12022-007-9001-3

[27] Takahashi Y, Onda M, Tanaka N, Seya T (2000) Establishment and characterization of two new rectal neuroendocrine cell carcinoma cell lines. Digestion 62: 262–270. 11070410 10.1159/000007825

[28] Kolby L, Bernhardt P, Ahlman H, Wangberg B, Johanson V, et al (2001) A transplantable human carcinoid as model for somatostatin receptor-mediated and amine transporter-mediated radionuclide uptake. Am J Pathol 158: 745–755. 11159212 10.1016/S0002-9440(10)64017-5 PMC1850312

[29] Van Buren G 2nd, Rashid A, Yang AD, Abdalla EK, Gray MJ, et al (2007) The development and characterization of a human midgut carcinoid cell line. Clin Cancer Res 13: 4704–4712. 17699847 10.1158/1078-0432.CCR-06-2723

[30] Grozinsky-Glasberg S, Shimon I, Rubinfeld H (2012) The role of cell lines in the study of neuroendocrine tumors. Neuroendocrinology 96: 173–187. 22538498 10.1159/000338793

[31] Luca AC, Mersch S, Deenen R, Schmidt S, Messner I, et al (2013) Impact of the 3D microenvironment on phenotype, gene expression, and EGFR inhibition of colorectal cancer cell lines. PLoS One 8: e59689. 23555746 10.1371/journal.pone.0059689 PMC3608563

[32] Krausch M, Kroepil F, Lehwald N, Lachenmayer A, Schott M, et al. (2013) Notch 1 tumor expression is lacking in highly proliferative pancreatic neuroendocrine tumors. Endocrine. 10.1007/s12020-012-9850-5 23225326

[33] Kenny PA, Lee GY, Myers CA, Neve RM, Semeiks JR, et al (2007) The morphologies of breast cancer cell lines in three-dimensional assays correlate with their profiles of gene expression. Mol Oncol 1: 84–96. 18516279 10.1016/j.molonc.2007.02.004 PMC2391005

[34] Pearse AG (1969) The cytochemistry and ultrastructure of polypeptide hormone-producing cells of the APUD series and the embryologic, physiologic and pathologic implications of the concept. J Histochem Cytochem 17: 303–313. 4143745 10.1177/17.5.303

[35] Rindi G, Bordi C (2005) Endocrine tumours of the gastrointestinal tract: aetiology, molecular pathogenesis and genetics. Best Pract Res Clin Gastroenterol 19: 519–534. 16183525 10.1016/j.bpg.2005.03.005

[36] Modlin IM, Lye KD, Kidd M (2003) A 5-decade analysis of 13,715 carcinoid tumors. Cancer 97: 934–959. 12569593 10.1002/cncr.11105

[37] Zhao CM, Jacobsson G, Chen D, Hakanson R, Meister B (1997) Exocytotic proteins in enterochromaffin-like (ECL) cells of the rat stomach. Cell Tissue Res 290: 539–551. 9369530 10.1007/s004410050960

[38] Eissele R, Anlauf M, Schafer MK, Eiden LE, Arnold R, et al (1999) Expression of vesicular monoamine transporters in endocrine hyperplasia and endocrine tumors of the oxyntic stomach. Digestion 60: 428–439. 10473967 10.1159/000007688

[39] Schuldiner S, Shirvan A, Linial M (1995) Vesicular neurotransmitter transporters: from bacteria to humans. Physiol Rev 75: 369–392. 7724667 10.1152/physrev.1995.75.2.369

[40] Erickson JD, Schafer MK, Bonner TI, Eiden LE, Weihe E (1996) Distinct pharmacological properties and distribution in neurons and endocrine cells of two isoforms of the human vesicular monoamine transporter. Proc Natl Acad Sci U S A 93: 5166–5171. 8643547 10.1073/pnas.93.10.5166 PMC39426

[41] Weihe E, Schafer MK, Erickson JD, Eiden LE (1994) Localization of vesicular monoamine transporter isoforms (VMAT1 and VMAT2) to endocrine cells and neurons in rat. J Mol Neurosci 5: 149–164. 7654518 10.1007/BF02736730

[42] James R, Erler T, Kazenwadel J (1994) Structure of the murine homeobox gene cdx-2. Expression in embryonic and adult intestinal epithelium. J Biol Chem 269: 15229–15237. 7910823

[43] Zhao J, de Krijger RR, Meier D, Speel EJ, Saremaslani P, et al (2000) Genomic alterations in well-differentiated gastrointestinal and bronchial neuroendocrine tumors (carcinoids): marked differences indicating diversity in molecular pathogenesis. Am J Pathol 157: 1431–1438. 11073802 10.1016/S0002-9440(10)64780-3 PMC1885722

[44] Lollgen RM, Hessman O, Szabo E, Westin G, Akerstrom G (2001) Chromosome 18 deletions are common events in classical midgut carcinoid tumors. Int J Cancer 92: 812–815. 11351300 10.1002/ijc.1276

[45] Terris B, Meddeb M, Marchio A, Danglot G, Flejou JF, et al (1998) Comparative genomic hybridization analysis of sporadic neuroendocrine tumors of the digestive system. Genes Chromosomes Cancer 22: 50–56. 9591634 10.1002/(sici)1098-2264(199805)22:1<50::aid-gcc7>3.0.co;2-6

[46] Tonnies H, Toliat MR, Ramel C, Pape UF, Neitzel H, et al (2001) Analysis of sporadic neuroendocrine tumours of the enteropancreatic system by comparative genomic hybridisation. Gut 48: 536–541. 11247899 10.1136/gut.48.4.536 PMC1728244

[47] Posorski N, Kaemmerer D, Ernst G, Grabowski P, Hoersch D, et al (2011) Localization of sporadic neuroendocrine tumors by gene expression analysis of their metastases. Clin Exp Metastasis 28: 637–647. 21681495 10.1007/s10585-011-9397-5

[48] Ellis LM, Samuel S, Sceusi E (2010) Varying opinions on the authenticity of a human midgut carcinoid cell line–letter. Clin Cancer Res 16: 5365–5366. 20959409 10.1158/1078-0432.CCR-10-2550

