# Supplementary figures and images for: New Model for Gastroenteropancreatic Large-Cell Neuroendocrine Carcinoma: Establishment of Two Clinically Relevant Cell Lines
Source: PLoS One. 2014 Feb 14;9(2):e88713. doi: 10.1371/journal.pone.0088713 (PMC3925161; doi:10.1371/journal.pone.0088713)

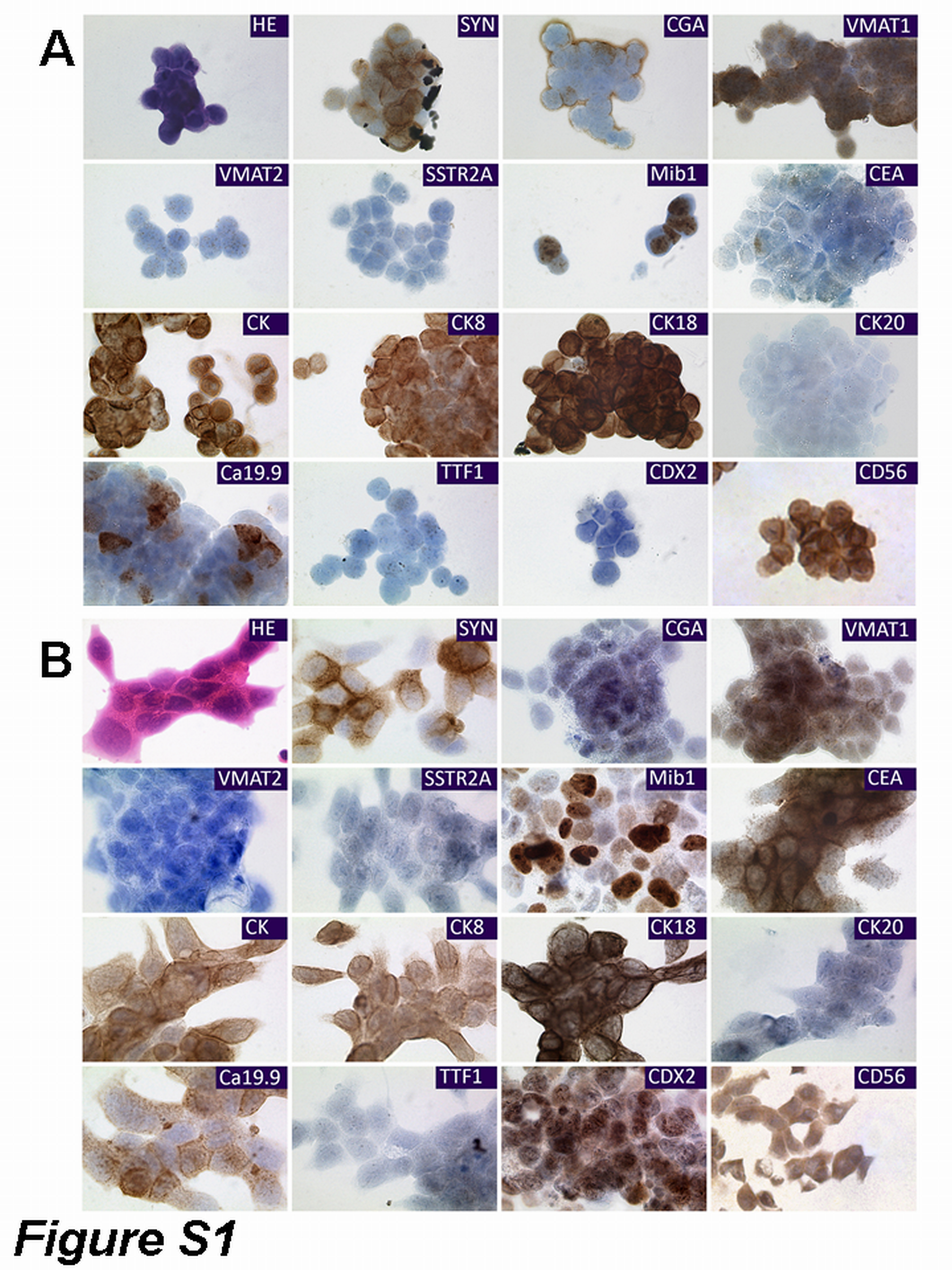

Supplement: Figure S1 — Immunocytochemical expression profile of NEC cell lines. NEC-DUE1 (A) and NEC-DUE2 (B) cells were grown on cover slips and stained with antibodies against general neuroendocrine markers, somatostatin receptors, proliferation marker, cytokeratines and epithelial markers as well as transcription factors. Abbreviations are explained in Table 1. (TIF) [file pone.0088713.s001.tif]
