# Supplementary material for: New Model for Gastroenteropancreatic Large-Cell Neuroendocrine Carcinoma: Establishment of Two Clinically Relevant Cell Lines
Source: PLoS One. 2014 Feb 14;9(2):e88713. doi: 10.1371/journal.pone.0088713 (PMC3925161; doi:10.1371/journal.pone.0088713)
Supplement: Table S1 — Antibodies used for immunocytochemistry and immunohistochemistry. (DOC) [file pone.0088713.s002.doc]

**Table S1: Antibodies used for immunocytochemistry and immunohistochemistry**

| **Antigen** | **Code** | **Source** | **Dilution** | **Species** |
| --- | --- | --- | --- | --- |
| ***General neuroendocrine markers*** |  |  |  |  |
| Synaptophysin | NCL-L-Synap 299 | Novocastra, Berlin, Germany | 1:100 | Mouse, mAK |
| Chromogranin A | MAB 5268 | Chemikon, Schwalbach, Germany | 1:1000 | Mouse, mAK |
| NSE | M0873 | Dako, Hamburg, Germany | 1:16000 | Mouse, mAK |
| VMAT 1 | H-V001 | Phoenix Pharmaceuticals, Inc., USA | 1:1500 | Rabbit, pAK |
| VMAT 2  CD56/NCAM | H-V003  IS628 | Phoenix Pharmaceuticals, Inc., USA  Dako, Hamburg, Germany | 1:1500  1:50 | Rabbit, pAK  Mouse, mAK |
| ***Somatostatin receptors*** |  |  |  |  |
| SSTR 2A | SS-800 | Gramsch Laboratories, Schwabhausen, Germany | 1:10 | Rabbit, pAK |
| SSTR 5 | SS-838 | Gramsch Laboratories, Schwabhausen, Germany | 1:5 / 1:2 | Rabbit, pAK |
| ***Proliferation*** |  |  |  |  |
| Ki-67 | M7240 | Dako, Hamburg, Germany | 1:500 | Mouse, mAK |
| ***Cytokeratins and epithelial markers*** |  |  |  |  |
| Pan-CK | MU071-UC | Biogenex, Fremont, CA, USA | 1:250 | Mouse, mAK |
| CK 8 | 503-3264 | Zytomed, Berlin, Germany | 1:100 | Rabbit, pAK |
| CK 18 | MU143-UC | Biogenex, Fremont, CA, USA | 1:2500 | Mouse, mAK |
| CK 20 | M7019 | Dako, Hamburg, Germany | 1:200 | Mouse, mAK |
| CEA | M7072 | Dako, Hamburg, Germany | 1:300 | Mouse, mAK |
| Ca 19.9 | NCL-Ca19-9 | Novocastra, Berlin, Germany | 1:600 | Mouse, mAK |
| ***Transcription factors*** |  |  |  |  |
| TTF1 | NCL-L-TTF | Novocastra, Berlin, Germany | 1:200 | Mouse, mAK |
| CDX2 | MU392A-UC | Biogenex, Fremont, CA, USA | 1:20 | Mouse, mAK |
