# Supplementary material for: New Model for Gastroenteropancreatic Large-Cell Neuroendocrine Carcinoma: Establishment of Two Clinically Relevant Cell Lines
Source: PLoS One. 2014 Feb 14;9(2):e88713. doi: 10.1371/journal.pone.0088713 (PMC3925161; doi:10.1371/journal.pone.0088713)
Supplement: Table S2 — Primers used for RT-PCR. (DOC) [file pone.0088713.s003.doc]

**Table S2: Primers used for RT-PCR**

| **Primer** | **Gene name** | **5`-3`Sequence** |
| --- | --- | --- |
| *CD56/NCAM*  forward | Neural cell adhesion molecule 1 | tac cgc ggc aag aac atc |
| *CD56/NCAM*  reverse |  | cca cct gca gag aaa ctg c |
| *CDX2* forward | Caudal type homeobox 2 | atc acc atc cgg agg aaa g |
| *CDX2* reverse |  | tgc ggt tct gaa acc aga tt |
| *CGA*  forward | Chromogranin A | gcg gtt ttg aag atg aac tct c |
| *CGA* reverse |  | gct ctt cca ccg cct ctt |
| *DDC* forward | Dopa decarboxylase | aag gag agt agc cct gta agg aa |
| *DDC* reverse |  | gta gtt ggc cac gta atc cac |
| *HDC* forward | Histidine decarboxylase | aac tgc ttg gga ttc acc tg |
| *HDC* reverse |  | cca acc agt cca tga cgt t |
| *ISL1* forward | Islet-1 | aag gac aag aag cga agc at |
| *ISL1* reverse |  | ttc ctg tca tcc cct gga ta |
| *NSE* forward | Neuron-specific enolase | act ttg tca ggg act atc ctg tg |
| *NSE* reverse |  | tcc cta cat tgg ctg tga act |
| *PGP9.5* forward | Protein gene product 9.5 | cct gaa gac aga gca aaa tgc |
| *PGP9.5* reverse |  | aaa tgg aaa ttc acc ttg tca tct |
| *SSTR2* forward | Somatostatin receptor 2 | gga gct agc gga ttg cag |
| *SSTR2* reverse |  | cag cca gcc cag aga tct ta |
| *SSTR5* forward | Somatostatin receptor 5 | gcg tca acc agt tca cca g |
| *SSTR5* reverse |  | gtg cac cac tgc cag gta |
| *SYN* forward | Synaptophysin | cca atc aga tgt agt ctg gtc agt |
| *SYN* reverse |  | agg cct tct cct gag ctc tt |
| *TH* forward | Tyrosine hydroxylase | gcc aag gac aag ctc agg |
| *TH* reverse |  | agc gtg tac ggg tcg aac t |
| *TPH1* forward | Tryptophan hydroxylase | ttg gaa gat gtc tcc aac ttt tta |
| *TPH1* reverse |  | aaa cct gat aag aaa tct ctt ggt g |
| *TTF1* forward | Transcription termination factor 1 | tca ttt gtt ggc gac tgg |
| *TTF1* reverse |  | tgc ttt gga ctc atc gac at |
| *VMAT1* forward | Vesicular monoamine transporter 1 | tcg tct atg ctc cac tct gc |
| *VMAT1* reverse |  | ttg cat aca tcc ggg tct c |
| *VMAT2* forward | Vesicular monoamine transporter 2 | cgg gat tct gca tca tgt tt |
| *VMAT2* reverse |  | tgg caa tca gca gga agg |
