# Supplementary material for: New Model for Gastroenteropancreatic Large-Cell Neuroendocrine Carcinoma: Establishment of Two Clinically Relevant Cell Lines
Source: PLoS One. 2014 Feb 14;9(2):e88713. doi: 10.1371/journal.pone.0088713 (PMC3925161; doi:10.1371/journal.pone.0088713)
Supplement: Table S3 — STR-analysis of established cell lines and corresponding primary tumors. (DOC) [file pone.0088713.s004.doc]

**Table S3: STR-analysis of established cell lines and corresponding primary tumors.**

| **STR Locus** | **NEC-DUE1 PT** | **Cell line** | **NEC-DUE2 PT** | **Cell line** |
| --- | --- | --- | --- | --- |
| **D8S1179** | 12-13 | 12-13 | 12-13 | 12-13 |
| **D21S11** | 32.2 | 32.2 | 28-29 | 29 |
| **D7S820** | 10-11 | 10-11 | 8-11 | 8-11 |
| **CSF1PO** | 12 | 12 | 13 | 13 |
| **D3S1358** | 16-17 | 16-17 | 15 | 15 |
| **TH01** | 6-9.3 | 6-9.3 | 8 | 8 |
| **D13S317** | 12 | 12 | 8-12 | 8-12 |
| **D16S539** | 10 | 10 | 10-11 | 10-11 |
| **D2S1338** | 19-20 | 19-20 | 17 | 17 |
| **D19S433** | 13-16 | 13-16 | 15-16 | 15-16 |
| **VWA** | 17 | 17 | 14-18 | 14 |
| **TPOX** | 8-9 | 8-9 | 8 | 8 |
| **D18S51** | 13-20 | 13-20 | 12-15 | 12-15 |
| **Amel** | XY | XY | X | X |
| **D5S818** | 13 | 13 | 12 | 12 |
| **FGA** | 20-25 | 20-25 | 21 | 21 |
| **D22S1045** | 15-16 | 15-16 | 11-16 | 11-16 |
| **D1S1656** | 14-15 | 14-15 | 12-14 | 12-14 |
| **D10S1248** | 16-17 | 16-17 | 14 | 14 |
| **D2S441** | 11 | 11 | 11 | 11 |
| **D12S391** | 18 | 18 | 17-23 | 17-23 |
| **SE33** | 25.2 | 25.2 | 22.2-24.2 | 22.2-24.2 |

STR short tandem repeat, PT primary tumor
